# Immune Biomarker Differences and Changes Comparing HCV Mono-Infected, HIV/HCV Co-Infected, and HCV Spontaneously Cleared Patients

**DOI:** 10.1371/journal.pone.0060387

**Published:** 2013-04-04

**Authors:** Lauren E. Kushner, Aaron M. Wendelboe, Laura C. Lazzeroni, Aarthi Chary, Mark A. Winters, Anu Osinusi, Shyam Kottilil, Michael A. Polis, Mark Holodniy

**Affiliations:** 1 Veterans Affairs Palo Alto Health Care System, Palo Alto, California, United States of America; 2 University of Oklahoma, Oklahoma City, Oklahoma, United States of America; 3 Stanford University, Stanford, California, United States; 4 Laboratory of Immunoregulation, National Institute of Allergy and Infectious Diseases, National Institutes of Health, Department of Health and Human Services, Bethesda, Maryland, United States of America; University of North Carolina School of Medicine, United States of America

## Abstract

**Background:**

Immune biomarkers are implicated in HCV treatment response, fibrosis, and accelerated pathogenesis of comorbidities, though only D-dimer and C-reactive protein have been consistently studied. Few studies have evaluated HIV/HCV co-infection, and little longitudinal data exists describing a broader antiviral cytokine response

**Methods:**

Fifty immune biomarkers were analyzed at baseline(BL) and HCV end of treatment follow-up(FU) time point using the Luminex 50-plex assay in plasma samples from 15 HCV-cleared, 24 HCV mono- and 49 HIV/HCV co-infected patients receiving antiretroviral treatment, who either did or did not receive pegylated-interferon/ribavirin HCV treatment. Biomarker levels were compared among spontaneous clearance patients, mono- and co-infected, untreated and HCV-treated, and sustained virologic responders (SVR) and non-responders (NR) at BL and FU using nonparametric analyses. A Bonferroni correction, adjusting for tests of 50 biomarkers, was used to reduce Type I error

**Results:**

Compared to HCV patients at BL, HIV/HCV patients had 22 significantly higher and 4 significantly lower biomarker levels, following correction for multiple testing. There were no significantly different BL levels when comparing SVR and NR in mono- or co-infected patients; however, FU levels changed considerably in co-infected patients, with seven becoming significantly higher and eight becoming significantly lower in SVR patients. Longitudinally between BL and FU, 13 markers significantly changed in co-infected SVR patients, while none significantly changed in co-infected NR patients. There were also no significant changes in longitudinal analyses of mono-infected patients achieving SVR or mono-infected and co-infected groups deferring treatment

**Conclusions:**

Clear differences exist in pattern and quantity of plasma immune biomarkers among HCV mono-infected, HIV/HCV co-infected, and HCV-cleared patients; and with SVR in co-infected patients treated for HCV. Though >90% of patients were male and co-infected had a larger percentage of African American patients, our findings may have implications for better understanding HCV pathogenesis, treatment outcomes, and future therapeutic targets

## Introduction

Inflammatory cytokines and chemokines have become increasingly important in the study of chronic human immunodeficiency virus (HIV) and hepatitis C virus (HCV) infection. As signaling molecules extensively involved in the immune system, cytokines and chemokines are vital for activating an effective immune response and recruiting immune cells to the site of infection. However, over- stimulation of the immune system can disrupt the pro-inflammatory/anti-inflammatory cytokine balance and have negative physiological effects.

Chronic HIV and/or HCV infection, microbial translocation, and opportunistic infections result in persistent immune activation that can accelerate the pathogenesis of HCV [1]–[5]. For example, liver damage and fibrosis commonly seen in HCV infection are immune-mediated processes resulting from chronic inflammation, and studies have shown that these processes are heightened in cases of HIV/HCV co-infection [6]–[9]. Several pro-inflammatory cytokines have also been associated with comorbidities such as vasculitis, atherosclerosis, and cardiovascular disease (CVD) in HIV-infected patients [5], [10]–[18]. It is therefore clinically important to understand the differential cytokine profiles of patients infected with either HCV alone or with both HIV and HCV.

Given their extensive role in immune activation and inflammation, cytokines have the potential to serve as biomarkers for HIV and HCV pathogenesis. Just as C-reactive protein (CRP) is used clinically to assess CVD risk and HIV disease progression respectively, monitoring levels of specific cytokines and mediators in HIV and HCV patients could predict their risk for developing comorbidities or their response rates to HCV treatment. Some existing biochemical markers, including alanine transaminase (ALT), aspartate transaminase (AST), and platelet counts, are already used to determine fibrosis scores and predict therapeutic outcomes for HCV patients [19], but further refinement and exploration of additional cytokine markers is warranted.

Early investigations consistently studied only the key mediators of inflammation – IL-1, IL-6, TNF-α, and CRP – in HIV and HCV mono-infected patients. While some recent studies have investigated additional cytokines with regard to either HIV/HCV co-infection or HCV treatment response, few have analyzed a broad spectrum of cytokines. To our knowledge, there is no comprehensive analysis of cytokine profiles that accounts for co-infection status, HCV treatment outcome, and spontaneous clearance of HCV. Thus, further investigation of cytokine dynamics is required to understand the impact of HIV/HCV co-infection and HCV treatment on the inflammatory response. Herein, we quantified and examined the levels of 50 plasma immune biomarkers among HCV mono-infected, HIV/HCV co-infected, and HCV spontaneous clearance patients, and compared levels both longitudinally and cross-sectionally across patient groups, which were further stratified by HCV treatment status and response.

## Materials and Methods

### Ethics Statement

The study was approved by Stanford University and NIH institutional review boards and was conducted under guidelines established by the Declaration of Helsinki. Written informed consent was obtained from all patients, and only patients who received conventional HCV treatment (weekly peg-interferon alfa-2a or 2b with weight based ribavirin) were included in this study. The two NIH clinical trials were registered in Clinicaltrails.gov (NCT00085917 and NCT00018031).

### Cohorts and Design

We performed both a cross-sectional and longitudinal study of HIV/HCV co-infected and HCV mono-infected patients from the National Institutes of Health (NIH) Clinical Research Center, Bethesda, MD and the Veterans Affairs Palo Alto Health Care System (VAPAHCS). HCV treatment outcomes were defined as sustained virologic response (SVR), rebound/relapse or non-response (NR). SVR patients were defined as having an undetectable HCV viral load up to 24 weeks after completing therapy. In contrast, patients who had a NR did not have at least a 2 log_10_ IU/mL drop in HCV viral load by week twelve of treatment. Rebound or relapse patients initially had a response to treatment (at least a 2 log_10_ IU/mL drop, though some achieved an undetectable HCV viral load), but then had breakthrough either during or after treatment, respectively, and failed to achieve SVR. For comparison purposes, rebound/relapse patients were grouped with those who experienced NR.

Patients were organized into groups based on their infection status and treatment status. The first group consisted of 38 HIV/HCV co-infected NIH Clinical Research Center patients who began an HCV regimen of weight-based ribavirin and pegylated interferon-α-2a or 2b. This group segregated into two subgroups by treatment outcome: 18 patients who eventually achieved SVR (co-infected sustained virologic responders, C-SVR) and 20 patients who experienced rebound/relapse or NR (co-infected non-responders, C-NR). Another co-infected group, co-infected deferring treatment (CDT), was comprised of eleven HIV/HCV co-infected VAPAHCS patients who were naïve to HCV treatment and did not begin HCV treatment at any point during the study. All co-infected patients in our study were on either HIV-1 protease inhibitor (PI)-based or non-nucleoside reverse transcriptase inhibitor (NNRTI)-based HIV antiretroviral therapy (ART), together with 2 HIV nucleoside reverse transcriptase inhibitors (NRTIs).

In addition to co-infected patients, the present study included two groups of HCV mono-infected patients. The first mono-infected group consisted of 13 VAPAHCS patients who were HCV treatment naïve and initiated ribavirin and pegylated interferon-α therapy (mono-infected starting treatment, MST), while the second mono-infected group consisted of 11 VAPAHCS patients who were not on nor planned to start HCV treatment (mono-infected deferring treatment, MDT). Finally, there was an additional control group of 15 VAPAHCS patients who were exposed to HCV but spontaneously cleared the virus. None of these control patients were HIV positive, and their exposure to HCV was confirmed by a positive HCV recombinant immunoblot assay and negative HCV viral load assay. For both co-infected and mono-infected patients, the untreated groups were not clinically different from their respective treatment group in terms of age, race, or gender. The decision to defer treatment depended on patient preference, as well as prognostic factors that could affect adherence.

### Descriptive and Clinical Patient Information

Patient medical records were used to obtain additional information on body mass index (BMI), medication use, and concurrent medical conditions. Laboratory results for ALT, AST, and platelet levels at BL and white blood cell (WBC) counts and differentials at BL and FU were also gathered for each patient. If BL lab results were not available on the exact day patients began the study, then the most recent lab results were used instead. However, lab results from dates after BL were not considered for any patient who began HCV treatment.

To assess stage of liver disease, a FIB-4 score was calculated using each patient’s age, AST and ALT levels, and platelet count. Per the correlation established by Vallet-Pichard et al. [19], any patient with a FIB-4 score greater than 3.25 was considered to have significant fibrosis comparable to a FibroTest score of F3–F4.

An age-adjusted Charlson Comorbidity Index score was also calculated for each person [20]. All patients in the C-SVR, C-NR, MST, and MDT groups, except for those with a FIB-4 score greater than 3.25, were identified as having mild liver disease based on the fact that they were all chronically infected with HCV. Those with a FIB-4 score greater than 3.25 were identified as having moderate to severe liver disease. Other conditions identified in one or more patients included diabetes, chronic obstructive pulmonary disease, connective tissue disease, peripheral vascular disease, lymphoma, any tumor, myocardial infarction, and congestive heart failure.

### Sample Preparation and Viral Load

Patients had their blood drawn at entry and again every four to eight weeks thereafter. HCV spontaneous clearance control patients only had blood drawn once. We selected two time points for evaluation: baseline (BL) and a follow-up (FU) time point, which for HCV-treated patients was no more than eight weeks after completing HCV treatment (Range: week 24–56). It is important to note that although a designation of SVR was used to categorize patients within HCV treatment groups, FU samples for all patients were taken before the six-month post-treatment time point and is therefore indicative of cytokine profiles at the end of treatment. In contrast, treatment outcome status was determined by appropriately drawn HCV viral loads done at the time of treatment failure (for rebound/relapse and NR) or at six months after treatment completion (for SVR). All HIV-1 and HCV plasma viral loads were determined using the Abbott RealTime HIV-1 and HCV assays according to manufacturer recommendations (Abbott Laboratories, Abbott Park, IL). CD4+ T cell counts were determined using standard flow cytometry assays. The plasma samples collected for each patient were aliquoted and stored at −70°C until used for analysis.

### Cytokine Analysis

Plasma samples were assayed using the Luminex 200 IS System according to manufacturer’s guidelines at the Human Immune Monitoring Center (Stanford, CA. http://iti.stanford.edu/research/documents/LuminexMultiplexAnalysisprotocol030911.doc). All samples were assayed for the following 50 pro-inflammatory plasma immune markers: CXCL5 (ENA-78), CCL11 (eotaxin), FGF, G-CSF, GM-CSF, CXCL1 (GRO-α), HGF, IFN-α, IFN-β, IFN-γ, IL-10, IL-12p40, IL-12p70, IL-13, IL-15, IL-17A, IL-17F, IL-18, IL-1α, IL-1β, IL-1RA, IL-2, IL-4, IL-5, IL-6, IL-7, CXCL8 (IL-8), CXCL10 (IP-10), leptin, LIF, CCL2 (MCP-1), CCL7 (MCP-3), M-CSF, MIG, CCL3 (MIP-1α), CCL4 (MIP-1β), NGF, PAI-1, PDGF-ββ, CCL5 (RANTES), resistin, SCF, sFasL, sICAM-1, sVCAM-1, TGF-α, TGF-β, TNF-α, TNF-β, and VEGF (Appendix S1). All samples were run in duplicate, and the average of duplicate measures was used for analysis. Values were reported in picograms per milliliter.

### Statistical Analysis

For participants whose HCV viral load fell to undetectable levels at FU, the midpoint between 0 and the lowest detectable limit was imputed. For participants whose cytokine concentrations were below the quantifiable limit (<x), a new value of (x-0.01) was imputed in order to carry out the analyses. To compare clinical and demographic variables, non-parametric statistical tests were conducted for non-normally distributed data (i.e., HCV viral loads), and parametric tests were conducted for normally distributed data (i.e., age and CD4 cell counts). Specifically the Kruskal-Wallis test was used for cross-sectional analyses to compare across groups at BL and FU. For the longitudinal analyses, the signed-rank test compared BL to FU values within groups. ANOVA was used when testing means across three groups (i.e., the HIV/HCV co-infected groups), and the Student’s t-test was used for comparing means across two groups (i.e., the HCV mono-infected groups). HIV viral load at both BL and FU was categorized into detectable versus non-detectable levels. For categorical data (i.e., sex, race, HCV genotype, and HIV viral load), the general association for comparison of proportions was calculated. For variables where there were no observations in a given cell, Fisher’s exact p-value was calculated.

Overall, we conducted tests of 50 biomarkers for each of 17 different comparisons or scientific hypotheses for a total of 850 tests. Simultaneous testing has the potential to inflate Type 1 error. However, multiple testing corrections necessarily inflate Type 2 error, increasing the chance of false negative conclusions. To balance the risks of Type 1 and Type 2 error, we applied a within-comparison Bonferroni correction, using an α = 0.05/50 = 0.001 significance level for all biomarker analyses. Whether to correct across distinct scientific hypotheses is a matter of controversy [21]–[24]. We note that the 0.001 significance level guarantees that, within a 50-test comparison, the probability of zero false positives is greater than 95%. Furthermore, we expect at most one false positive in 1,000 tests. For the total 850 tests conducted, this amounts to less than one false positive (0.001 × 850 = 0.85). Using a more stringent significance level of 0.001/17 = 0.0006 to correct across comparisons would have reduced the overall expected number of false positives to at most 0.05 (a reduction of less than 1 false positive), while increasing the expected number of false negatives by an unknown number. For those biomarkers satisfying the 0.001 significance level, we considered the possibility that the results could be confounded by certain clinical factors. To examine this question, we used logistic regression with an outcome defined as a biomarker level above or below the median for that cytokine. We adjusted this model for BMI, FIB-4 score, age-adjusted Charlson score, and use of specific medications (statins, NSAIDS, and other steroids).

## Results

### Descriptive and Clinical Characteristics

Forty-nine HIV/HCV co-infected, 24 HCV mono-infected, and 15 HCV spontaneous clearance patients were included in the analysis. Table 1 outlines the demographic information for the patients in the study. The majority of patients were male (83 [94%] of 88 patients). Of the 88 patients, 32 [36%] were African American, 44 [50%] were Caucasian, ten [11%] were Hispanic, one [1%] was Native American, and one [1%] was Asian/Pacific Islander. The median age for patients at BL was 52 years. There were no significant differences between C-SVR and C-NR in terms of sex, age, race, HCV viral load, or genotype. There were also no significant differences in sex or genotype between mono-infected and co-infected patients. However, mono-infected patients were significantly older than co-infected patients (median age: 54.5 vs. 49 years, p = 0.004); in addition, the majority of mono-infected patients were Caucasian (70.8%), whereas the majority of co-infected patients were African American (55.1%) and only 34.7% were Caucasian (p<0.001). BL HCV viral load was not significantly different between mono-infected and co-infected patients (p = 0.24); however, among the co-infected groups, the C-SVR group did have significantly lower BL median HCV viral load when compared to all other co-infected patients, including both C-NR and CDT groups combined (p = 0.03).

**Table 1:** Summary of Patient Demographics.

	HIV/HCV Co-infected				HCV Mono-infected			Spontaneously Cleared HCV
Characteristic	C-SVR (n = 18)	C-NR (n = 20)	CDT (n = 11)	p	MST (n = 13)	MDT (n = 11)	p	(n = 15)
Mean age (range), years	46 (33–60)	48 (33–64)	54 (49–58)	0.008	55 (49–62)	54 (46–61)	0.54	55 (48–63)
Male sex (%)	16 (89%)	18 (90%)	11 (100%)	0.54	12 (92%)	11 (100%)	0.54*	15 (100%)
Race				0.55			0.52	
African American	7 (39%)	12 (60%)	8 (73%)		1 (8%)	3 (27%)		1 (6.7%)
Asian/Pacific Islander	1 (6%)	0 (0%)	0 (0%)		0 (0%)	0 (0%)		0 (0%)
Caucasian	8 (44%)	7 (35%)	2 (18%)		10 (77%)	7 (64%)		10 (66.7%)
Hispanic	2 (11%)	1 (5%)	1 (9%)		1 (8%)	1 (9%)		4 (26.7%)
Native American	0 (0%)	0 (0%)	0 (0%)		1 (8%)	0 (0%)		0 (0%)
Median BMI (range), kg/m^2^	26 (21–32)	25.5 (21–38)	26 (20–30)	0.75	30 (22–38)	30 (24–36)	0.71	27 (19–39)
Mean FIB-4 Index (range)	1.6(0.63–5.94)	1.95(0.73–4.45)	3.71(0.99–13.44)	0.02	3.02(0.54–7.16)	1.35(0.64–2.05)	0.017	1.78(0.75–7.34)
Median Age Adjusted Charlson ComorbidityIndex Score (range)	1 (1–6)	1.5 (1–6)	3 (1–7)	0.002	2 (1–5)	2 (1–4)	0.81	0 (0–4)
Other Medication Use During Study								
Statins (Yes, No)	0, 17	0, 19	1, 10	0.195	0, 13	4, 7	0.020	3, 12
NSAIDs (Yes, No)	12, 5	18, 1	1, 10	<0.001	3, 10	3, 8	0.817	3, 12
Other Steroid‡ (Yes, No)	1, 16	4, 15	1, 10	0.371	0, 13	0, 11	–	1, 14
Mean White Blood Cell Count, cells/µL								
Baseline ×10^3^ (range)	5.5 (2.5–10.2)	5.3 (3.2–11.9)	4.5 (3.0–7.7)	0.32	6.6 (0.8–12.1)	7.2 (4.8–9.8)	0.53	7.6 (4.1–12.5)
Follow-up ×10^3^ (range)	4.7 (2.7–8.7)	4.6 (2.0–8.5)	4.1 (2.1–7.3)	0.60	4.8 (2.4–10.3)	7.1 (4.5–9.4)	0.02	–
Mean Percentage of Neutrophils, %								
Baseline (range)	51.3 (32.3–73.4)	42.7 (28.9–71.1)	53.3 (38.0–71.5)	0.05	56.3 (41.7–70.2)	62.0 (39.5–73.4)	0.13	63.7 (52.9–74.2)
Follow-up (range)	54.1 (38.2–73.3)	44.2 (16.5–62.0)	53.3 (40.1–69.5)	0.02	60.4 (49.3–73.8)	61.3 (49.1–71.1)	0.84	–
HCV Genotype				0.67			0.82	
1	13 (72%)	17 (85%)	8 (73%)		7 (53.8%)	7 (64%)		–
2	4 (22%)	2 (10%)	1 (9%)		4 (30.8%)	2 (18%)		–
3	0	0	1 (9%)		1 (7.7%)	2 (18%)		–
4	0	1 (5%)	0		1 (7.7%)	0		–
5	1 (6%)	0	0		0	0		–
1/4	0	0	1 (9%)		0	0		–
Median HCV RNA level, IU/mL								
Baseline×10^3^	410	1954	1690	0.03	1640	4160	0.58	–
(range)	2–8300	648–12190	182–5610		87–7240	107–20000		
Follow-up×10^3^	und^1^ †	717	1940	0.20	2375	2970	0.01	–
(range)	–	und^1^ – 16035	83 – >37000		und^2^ – >8000	193–>8000		
Median HCV treatment duration (range), weeks	48 (18–48)	48 (15–72)	–	–	26 (3–48)	–	–	–
Median CD4^+^ cell count (range), cells/mL								
Baseline	501 (127–897)	514 (108–1273)	365 (94–885)	0.17	–	–	–	–
Follow-up	353 (52–640)	409 (120–1123)	247 (125–568)	0.27	–	–	–	–
Undetectable HIV RNA level (%)								
Baseline	10 (56%)	16 (80%)	11 (100%)	0.02	–	–	–	–
Follow-up	14 (78%)	15 (75%)	11 (100%)	0.36	–	–	–	–

Note: Data listed as total number of patients, unless otherwise noted. C-SVR: co-infected sustained virologic responders; C-NR: co-infected non-responders; CDT: co-infected deferring treatment; MST: mono-infected starting treatment; MDT: mono-infected deferring treatment; BMI: body mass index; NSAID: Non-Steroidal Anti-inflammatory drug; und: undetectable or below the lower limit of detection (LLOD).

1 LLOD is <615 IU/mL;

2 LLOD is <20 IU/mL.

† One patient in this group had an HCV RNA level of 673 IU/mL at follow-up, but still achieved SVR.

‡ Excludes steroids administered topically or intranasally.

* Fisher’s exact p-value.

Liver disease status, as determined by FIB-4, and use of steroid medications did not significantly differ between mono-infected and co-infected patients. However, significantly more co-infected patients used non-steroidal anti-inflammatory drugs (NSAIDs) at the time of the study (p = 0.002), and more mono-infected patients were on statin medications (p = 0.09). Mono-infected patients also had a significantly increased comorbidity risk, as determined by Charlson score (p = 0.017). C-SVR and C-NR groups did not significantly differ in terms of FIB-4, Charlson score, statin use, or steroid medication use. However, significantly more C-NR patients used NSAIDs (p = 0.04).

Of the 38 co-infected patients who began HCV treatment, 18 achieved SVR, whereas the remaining 20 either had a non-response (n = 5), relapse (n = 10), or rebound (n = 5). Of the thirteen HCV mono-infected patients who began HCV treatment, six achieved SVR, but the remaining seven either relapsed (n = 1), discontinued treatment (n = 4), or dropped out of the study (n = 2). For the four patients who discontinued treatment during the course of the study, three discontinuations were provider-driven, while the fourth patient discontinued voluntarily due to intolerance of treatment side effects. Notably, only one of the four treatment discontinuation patients had shown a response to treatment by week twelve. Discontinuation and dropout patients were considered as non-responders.

The median duration of treatment was 48 weeks for co-infected patients (C-SVR and C-NR) and 24 weeks for the HCV mono-infected patients (MST) (p<0.001). The significant variation in treatment duration can be explained by the predominance of genotype 1 among co-infected patients (79% among those receiving treatment) and by current guidelines recommending 48 weeks of HCV treatment for most co-infected patients. Only 54% of MST patients were genotype 1. Nonetheless, the MST group also had a higher rate of discontinuation and drop out than the NIH co-infected population, which also contributed to the disparity in treatment duration. Mean white blood cell count (WBC) was not significantly different at baseline or at end of treatment in C-SVR and C-NR compared to CDT, whereas WBC was significantly decreased in MST at end of treatment compared to MDT (p = 0.02). Although neutrophil percentage was significantly lower in C-NR compared to C-SVR and CDT at baseline and follow-up (p<0.05), there was no significant decrease in neutrophil percentage in C-NR after HCV treatment. There were no significant differences in MST or MDT neutrophil percentage.

### Biomarkers

**Figure 1:**
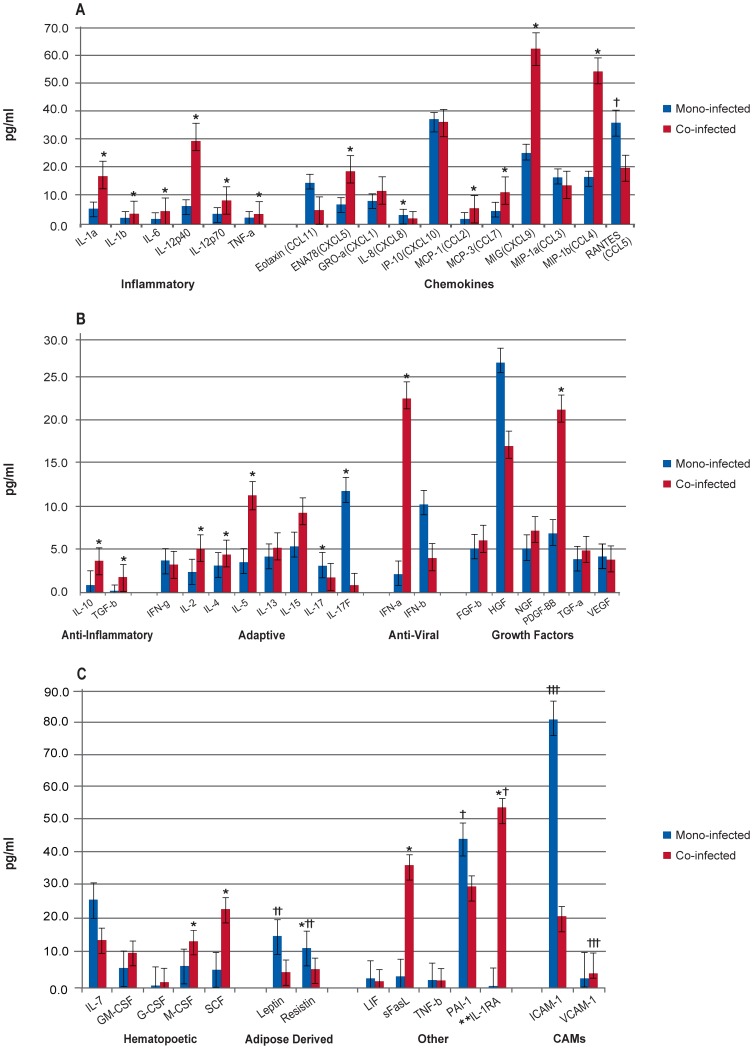
Comparison of HCV Mono-infected and HCV/HIV Co-infected Patients at Baseline. Median biomarker levels (pg/ml) are divided into 3 panels A) Inflammatory, chemokines; B) anti-inflammatory, adaptive, anti-viral, growth factors; and C) hematopoietic, adipose-derived, other, and cellular adhesion molecular (CAMs). Blue bar = HCV mono-infected (Mono-infected), red bar = HCV/HIV co-infected (Co-infected). Concentrations are actual values, unless otherwise noted (†actual values 10x, ††actual values 100x, and †††actual values 1000x). *p<0.001. **IL-1RA is “anti-inflammatory”, but graphed with “Others” for scale.

A summary of biomarker results is presented in Table 2. When comparing all HIV/HCV co-infected and HCV mono-infected patients at BL, the HIV/HCV co-infected patients had significantly higher levels of 22 cytokines (IL-1α, IL-1β, IL-6, IL-12p40, IL-12p70, TNF- α, IL-RA, IL-10, TGF- β, IFN- α, ENA78, MCP-1, MCP-3, MIG, MIP-1 β, PDGF-ββ,, M-CSF, SCF, IL-2, IL-4, IL-5, and sFasL). In contrast, four marker levels (IL-8, IL-17, IL-17F, and resistin) were significantly higher in the mono-infected patients (Figure 1, Appendix S2.a).

**Table 2:** Biomarkers of Significance Grouped by Comparisons between HCV and HIV/HCV Co-Infected and HCV Spontaneously Cleared Cohorts.

Cross-sectional Analyses	
Comparison	Cytokines of Significance
**Mono-infected** (n = 24) vs. **Co-infected** (n = 47) at BL	Higher in Mono-infected: IL-8, IL-17, IL-17F, and Resistin
	Higher in Co-infected: IL-1α, IL-1β, IL-6, IL-12p40, IL-12p70, TNF- α, IL-RA, IL-10, TGF- β, IFN- α, ENA78, MCP-1, MCP-3, MIG, MIP-1β, PDGF-ββ, M-CSF, SCF, IL-2, IL-4, IL-5, and sFasL
**MST-SVR** (n = 6) vs. **MST-NR** (n = 7) at BL	No Significant Differences
**C-SVR** (n = 18) vs. **C-NR** (n = 18) at BL	No Significant Differences
**C-SVR** (n = 18) vs. **C-NR** (n = 19) at FU	Higher in C-SVR: IL-8, MCP-1, MIP-1β, RANTES, PDGF-ββ, IL-7, and PAI-1
	Higher in C-NR: IL-1β, IL-12p40, IFN-α, TGF-α, M-CSF, SCF, sFasL, and TNF-β
**Combined-SVR** (n = 24) vs. **Spontaneous Clearance** (n = 15) at BL	Higher in Combined-SVR: IL-6, IL-12p40, IL-12p70, TNF-α, IFN-α, IFN-β, ENA78, IL-8, IP-10, MIP-1α, FGF-β, HGF, TGF-α, VEGF, IL-7, M-CSF, IFN-γ, IL-13, IL-17, IL-17F, VCAM-1, and TNF-β
**Combined-SVR** (n = 24) vs. **Spontaneous Clearance** (n = 15) at FU	Higher in Combined-SVR: IL-12p40, IL-12p70, IFN-α, IP-10, FGF-β, TGF-α, VEGF, IL-7, IL-13, IL-17, IL-17F, and TNF-β
**Longitudinal Analyses**	
**Comparison**	**Cytokines of Significance**
**MST-SVR:** BL (n = 6) vs. FU (n = 6)	No Significant Differences
**C-SVR:** BL (n = 18) vs. FU (n = 18)	Significant Decreases: IFN-α, M-CSF, ICAM-1, VCAM-1, sFasL, and TNF-β
	Significant Increases: IL-8, MCP-1, MIP-1β, RANTES, PDGF-ββ, IL-7, and PAI-1
**C-NR:** BL (n = 18) vs. FU (n = 19)	No Significant Differences
**CDT:** BL (n = 11) vs. FU (n = 8)	No Significant Differences
**MDT:** BL (n = 11) vs. FU (n = 10)	No Significant Differences

Note: Results for each analysis were considered statistically significant if p<0.001. BL: baseline; FU: follow-up; C-SVR: co-infected sustained virologic responders; C-NR: co-infected non-responders; CDT: co-infected deferring treatment; MST-SVR: mono-infected starting treatment with sustained virologic response; MDT: mono-infected deferring treatment.

**Figure 2:**
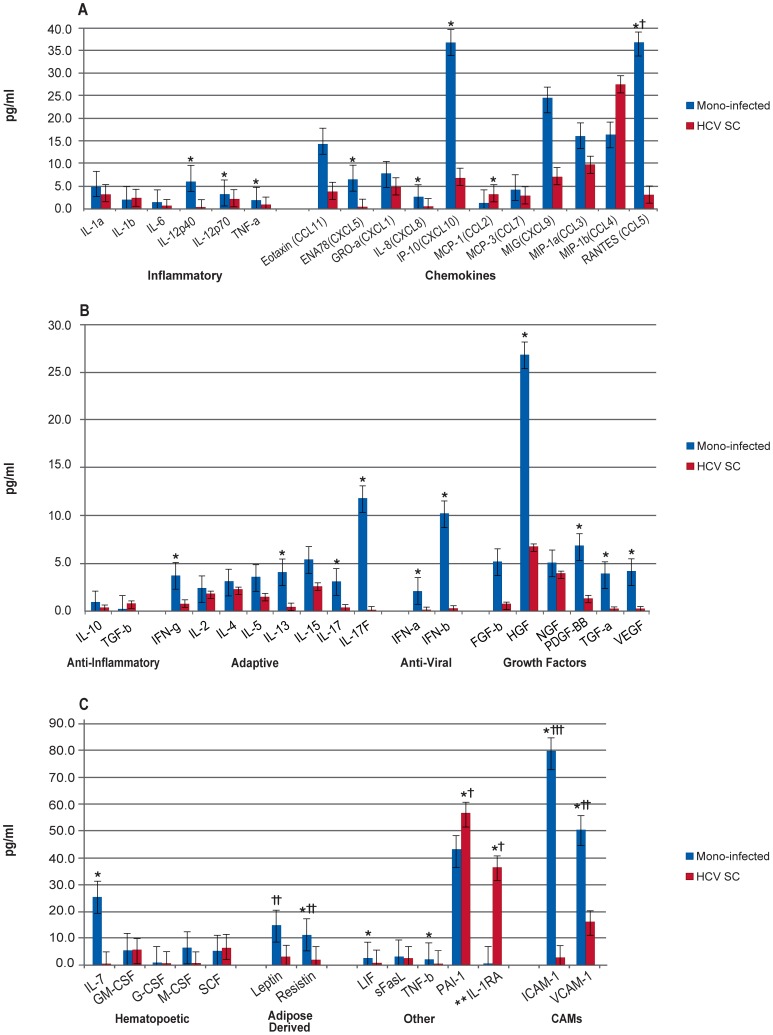
Comparison of HCV Mono-infected Patients and Patients who Spontaneously Cleared HCV Infection at Baseline. Median biomarker levels (pg/ml) are divided into 3 panels A) Inflammatory, chemokines; B) anti-inflammatory, adaptive, anti-viral, growth factors; and C) hematopoietic, adipose-derived, other, and cellular adhesion molecular (CAMs). Blue bar = HCV mono-infected (Mono-infected), red bar = spontaneously cleared HCV infection (HCV SC). Concentrations are actual values, unless otherwise noted (†actual values 10x, ††actual values 100x, and †††actual values 1000x). *p<0.001. **IL-1RA is “anti-inflammatory”, but graphed with “Others” for scale.

**Figure 3:**
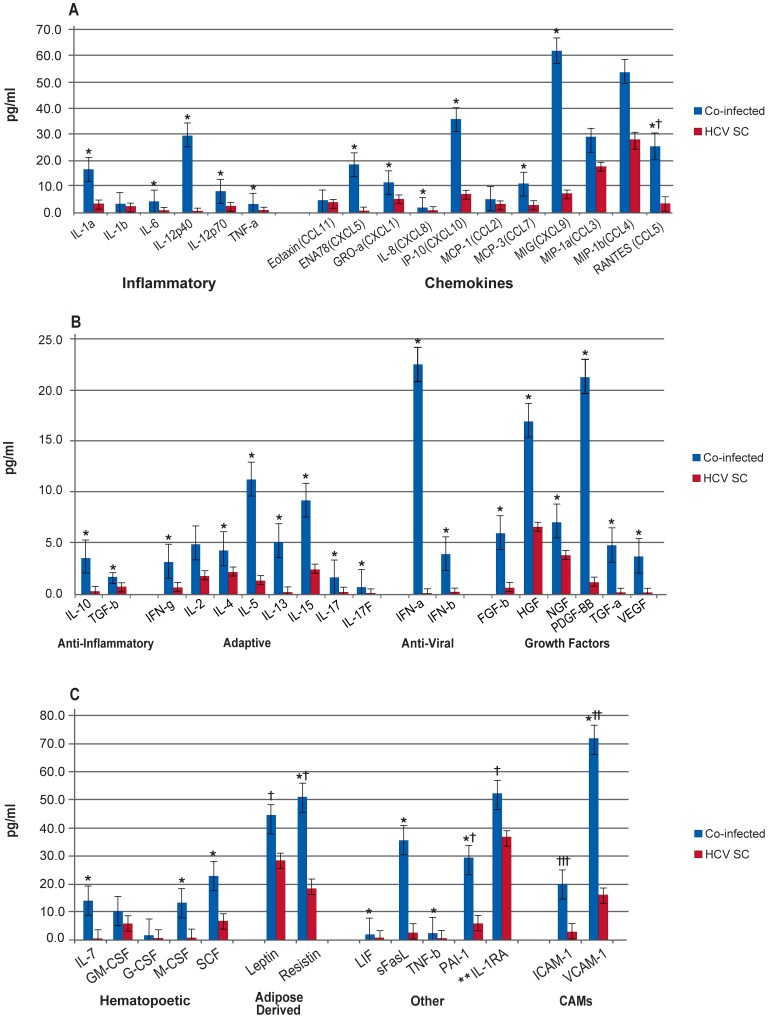
Comparison of HCV/HIV co-infected Patients and Patients who Spontaneously Cleared HCV Infection at Baseline. Median biomarker levels (pg/ml) are divided into 3 panels A) Inflammatory, chemokines; B) anti-inflammatory, adaptive, anti-viral, growth factors; and C) hematopoietic, adipose-derived, other, and cellular adhesion molecular (CAMs). Blue bar = HCV/HIV co-infected (Co-infected), red bar = spontaneously cleared HCV infection (HCV SC). Concentrations are actual values, unless otherwise noted (†actual values 10x, ††actual values 100x, and †††actual values 1000x). *p<0.001. **IL-1RA is “anti-inflammatory”, but graphed with “Others” for scale.

In a comparison of HCV mono-infected patients at BL to the HCV spontaneous clearance group (Figure 2, Appendix S2.b), the mono-infected group had significantly lower levels of IL-1RA and MCP-1 compared to the HCV spontaneous clearance group. Whereas, when the co-infected patients were compared to the HCV spontaneous clearance group at BL (Figure 3, Appendix S2.b), the majority of the biomarkers were significantly elevated in co-infected patients. Many of these biomarkers were also significantly elevated in the mono-infected patients compared to the HCV spontaneous clearance group, although the total number of significantly increased cytokines was much less than in the co-infected patients.

**Figure 4:**
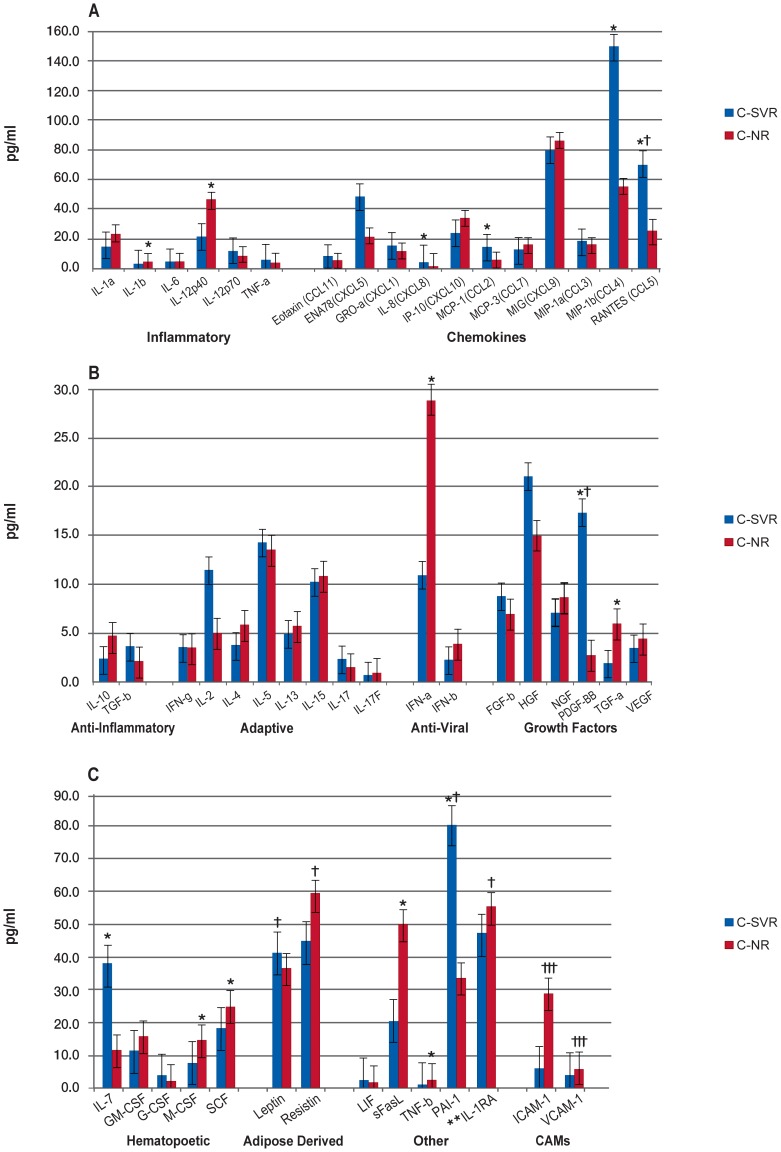
Comparison of HCV/HIV Co-infected Patients with Sustained Virologic Response (SVR) versus Nonresponse (NR) to HCV Treatment. Median biomarker levels (pg/ml) are divided into 3 panels A) Inflammatory, chemokines; B) anti-inflammatory, adaptive, anti-viral, growth factors; and C) hematopoietic, adipose-derived, other, and cellular adhesion molecular (CAMs). Blue bar = HCV/HIV co-infected SVR (C-SVR), red bar = HCV/HIV co-infected nonresponse (C-NR). Concentrations are actual values, unless otherwise noted (†actual values 10x and †††actual values 1000x). *p<0.001. **IL-1RA is “anti-inflammatory”, but graphed with “Others” for scale.

**Figure 5:**
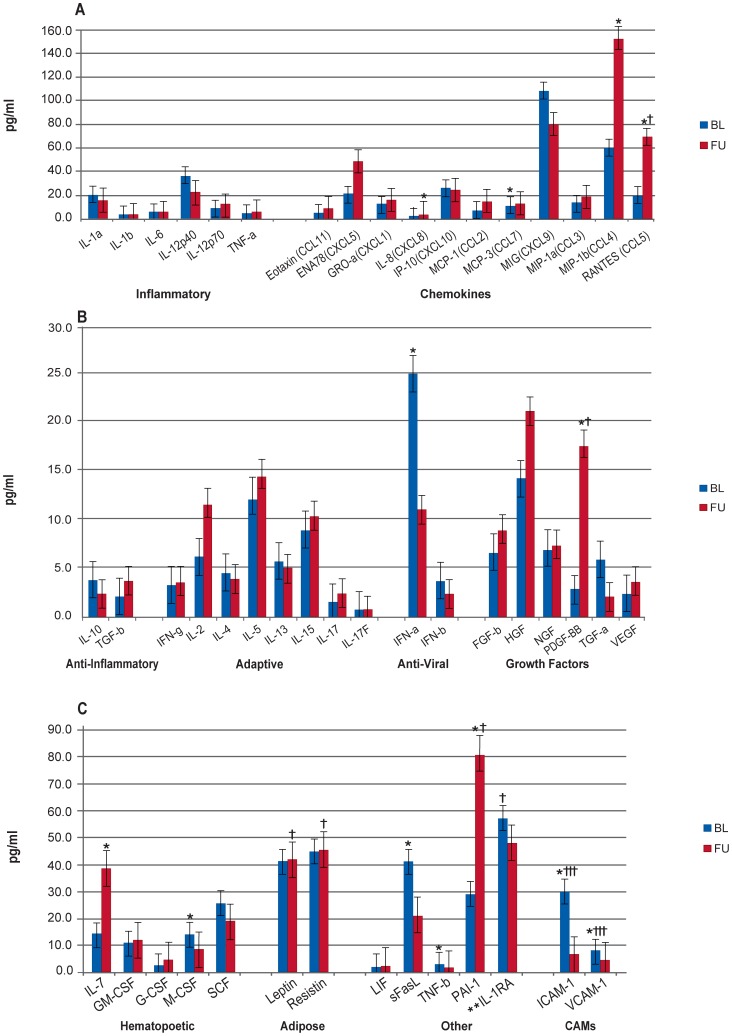
Comparison of Baseline and Follow-up in HCV/HIV Co-infected Patients with Sustained Virologic Response (SVR). Median biomarker levels (pg/ml) are divided into 3 panels A) Inflammatory, chemokines; B) anti-inflammatory, adaptive, anti-viral, growth factors; and C) hematopoietic, adipose-derived, other, and cellular adhesion molecular (CAMs). Blue bar = baseline (BL), red bar = follow-up (FU). Concentrations are actual values, unless otherwise noted (†actual values 10x and †††actual values 1000x). *p<0.001. **IL-1RA is “anti-inflammatory”, but graphed with “Others” for scale.

When analysis was restricted to co-infected patients, there were no significant BL differences in biomarker concentrations between C-SVR patients and C-NR patients (Appendix S3.a). In contrast, a comparison of C-SVR and C-NR patients at FU revealed that 7 biomarker levels (IL-8, MCP-1, MIP-1β, RANTES, PDGF-ββ, IL-7, and PAI-1) were significantly higher in C-SVR patients, while 8 marker levels (IL-1β, IL-12p40, IFN-α, TGF-α, M-CSF, SCF, sFasL, and TNF-β) were significantly higher in C-NR patients (Figure 4, Appendix S3.a). Finally, when comparing BL to FU values within the C-NR group, none of the 50 biomarkers differed significantly between the two time points (Appendix S3.b). However, in the C-SVR cohort, 7 biomarkers (IL-8, MCP-1, MIP-1β, RANTES, PDGF-ββ, IL-7, and PAI-1) increased significantly from BL to FU, while 6 markers (IFN-α, M-CSF, ICAM-1, VCAM-1, sFasL, and TNF-β) decreased significantly between BL and FU (Figure 5, Appendix S3.b). For the CDT group (Appendix S4), no biomarkers were significantly different between BL and FU.

Just as the C-SVR group did not significantly differ from the C-NR group at BL, the BL biomarker profiles of MST patients who achieved SVR did not differ significantly from those of the MST patients who had a NR (Appendix S5). Within the MST group, a BL to FU comparison (Appendix S6) for the six patients that achieved SVR indicated that no biomarkers significantly decreased with treatment between these two time points. Similarly in the MDT group, no biomarkers changed between BL and FU (Appendix S4).

**Figure 6:**
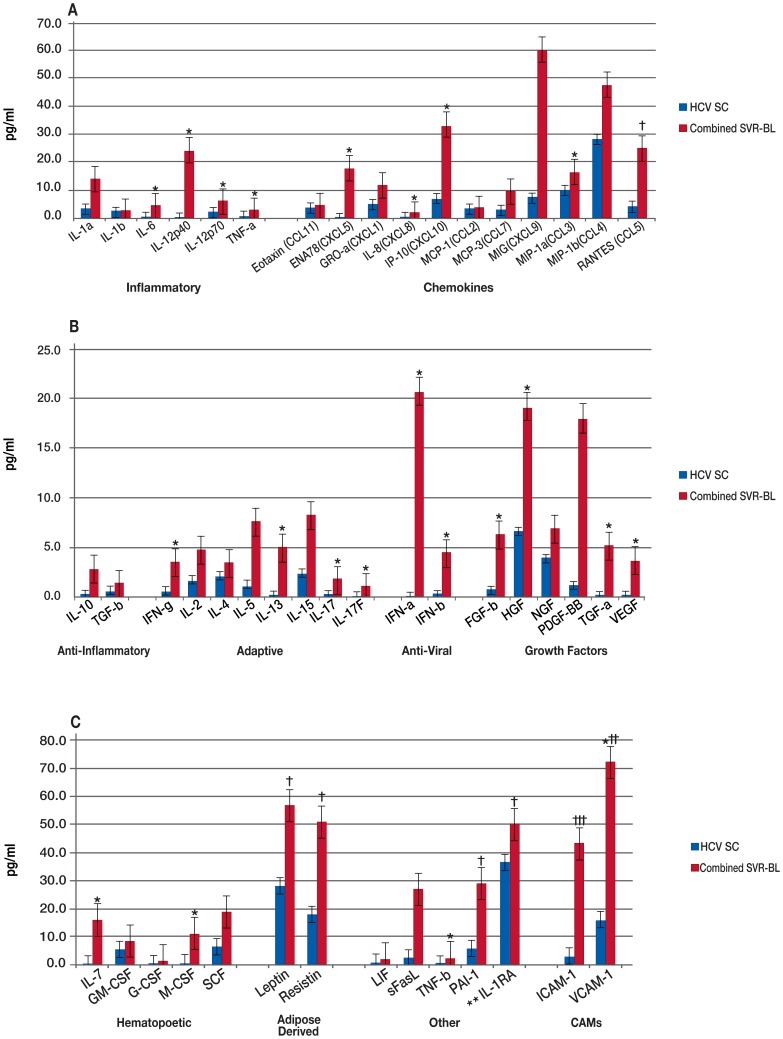
Comparison of Spontaneous Clearance Group to all Patients with Sustained Virologic Response (SVR) at Baseline. Median biomarker levels (pg/ml) are divided into 3 panels A) Inflammatory, chemokines; B) anti-inflammatory, adaptive, anti-viral, growth factors; and C) hematopoietic, adipose-derived, other, and cellular adhesion molecular (CAMs). Blue bar = HCV spontaneous clearance (HCV SC), red bar = HCV mono-infected and HIV/HCV co-infected patients with SVR (Combined SVR-BL). Concentrations are actual values, unless otherwise noted (†actual values 10x, ††actual values 100x and †††actual values 1000x). *p<0.001. **IL-1RA is “anti-inflammatory”, but graphed with “Others” for scale.

**Figure 7:**
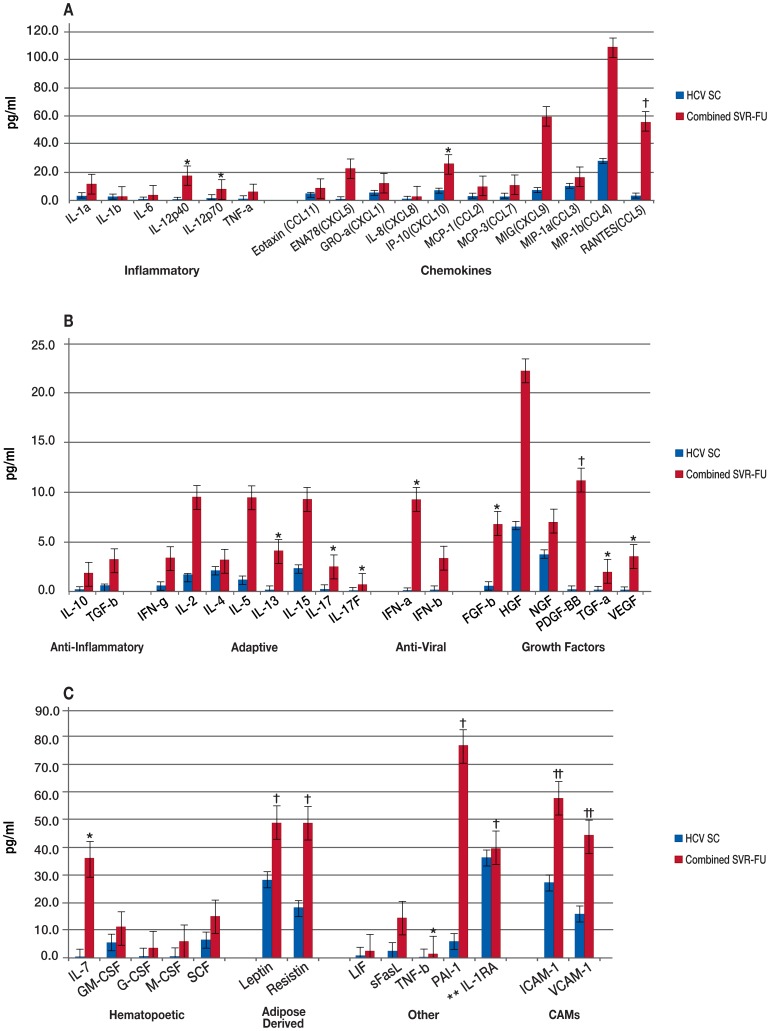
Comparison of Spontaneous Clearance Group to all Patients with Sustained Virologic Response (SVR) at Follow-up. Median biomarker levels (pg/ml) are divided into 3 panels A) Inflammatory, chemokines; B) anti-inflammatory, adaptive, anti-viral, growth factors; and C) hematopoietic, adipose-derived, other, and cellular adhesion molecular (CAMs). Blue bar = HCV spontaneous clearance (HCV SC), red bar = HCV mono-infected and HIV/HCV co-infected patients with SVR (Combined SVR-FU). Concentrations are actual values, unless otherwise noted (†actual values 10x, ††actual values 100x). *p<0.001. **IL-1RA is “anti-inflammatory”, but graphed with “Others” for scale.

In addition, we compared the HCV spontaneous clearance group to all patients who achieved SVR (both co-and mono-infected) at BL and FU (Figures 6 and 7, Appendix S7), initially comparing individual C-SVR and MST-SVR groups to the spontaneous clearance group (data not shown). Only biomarkers that were significantly elevated in the combined-SVR group and in both C-SVR and MST-SVR comparisons were considered. A total of 22 markers were significantly elevated in the combined-SVR group versus the spontaneous group at BL. Of these, 12 (IL-12p40, IL-12p70, IFN-α, IP-10, FGF-b, TGF-α, VEGF, IL-7, IL-13, IL-17, IL-17F, and TNF-β) remained significantly elevated at FU as well. In contrast, 10 (IL-6, TNF-α, IFN-β, ENA-78, IL-8, MIP-1α, HGF, M-CSF, IFN-γ, and VCAM-1) were significantly elevated at BL, but not at FU.

Finally, we compared our significant, non-parametric biomarker findings, to both unadjusted and adjusted parametric analyses, taking into account several possible confounding variables. The parametric models yielded qualitatively similar results to the non-parametric analyses; however, estimates became unstable when covariates were added, often resulting in substantially larger odds ratios and wider confidence intervals. We believe that the adjusted models were over parameterized given the small sample sizes, and so these results were not considered any further (data not shown).

## Discussion

We observed distinct differences among biomarker profiles of HCV mono-infected patients, HIV co-infected patients, and those who spontaneously cleared HCV infection. These observations can be summarized as two central conclusions. First, our study confirms that co-infected patients have significantly increased levels of pro-inflammatory cytokines compared to mono-infected patients and enhances the current understanding of this heightened inflammatory state by evaluating 50 different biomarkers. Secondly, though the cytokine profiles of C-SVR and C-NR patients did not significantly differ at BL, they differed considerably at FU after patients received 48 weeks of HCV treatment, and these cross-sectional differences were largely driven by longitudinal changes within the C-SVR group. To our knowledge, this is the first study of HCV mono-infected, HIV/HCV co-infected, and HCV spontaneous clearance patients to both evaluate and report the results of 50 different markers with regard to infection status and treatment outcome. Notably, over 90% of patients were male, sample sizes were small, and the HIV/HCV co-infected group had a larger percentage of African American patients than the HCV mono-infected group, all of which could affect immune marker differences between groups.

When compared to mono-infected patients at BL, HIV/HCV co-infected patients in our study had significantly higher levels of cytokines involved in inflammation (IL-1α, IL-1β, IL-6, IL-12p40, IL-12p70, and TNF-α), chemotaxis (ENA78, MCP-1, MCP-3, MIG, and MIP-1β), hematopoiesis (M-CSF, and SCF), and fibrosis (TGF-β, IL-4, and IL-5). This heightened inflammatory state may offer clues as to why conventional antiviral therapy for HCV infection may not be as effective or why prolonged treatment is necessary to achieve an SVR relative to mono-infected patients. This observation is further supported by comparisons of co-infected and mono-infected patients to those who spontaneously cleared HCV and therefore represent the baseline inflammatory profile of patients in the absence of chronic viral infection. Both mono-infected and co-infected groups had significantly elevated levels of many inflammatory markers over the spontaneous clearance group, though the differences were more pronounced in co-infected patients, further highlighting the increased inflammation in the co-infected population.

The co-infected cytokine profile appeared to favor a Th2 response by having increased IL-4, IL-5, and IL-10 and lacking a significant elevation in IFN-γ. Previous researchers have suggested that a greater Th2 response is established with chronic HCV infection and questioned whether it is a cause or result of the immune system’s inability to clear the viral infection [25]–[26]. It is known that a heightened Th2 response shifts the immune system away from a cellular immune response, which is necessary for controlling viremia and is mediated by Th1. Given that previous findings have associated Th2 responses with enhanced hepatic fibrosis [26]–[28], the significantly elevated Th2 cytokines IL-4 and IL-5 in co-infected patients may be a major reason that co-infected patients tend to have accelerated progression of HCV.

In addition to a prevalence of Th2 cytokines, our results indicated increased biomarker activity promoting hematopoiesis, chemotaxis, and inflammation in co-infected patients. Hematopoietic markers stimulate precursors in the bone marrow to develop into granulocytes and monocytes, while chemokines then recruit these neutrophils and monocytes to the site of infection, where they release additional cytokines that perpetuate the cytokine storm. Persistent activation and accumulation of these immune cells are a major source of reactive oxygen species, reactive nitrogen species, and pro-inflammatory cytokines, all of which contribute to tissue damage and likely facilitate accelerated pathogenesis of HCV in co-infected patients [5], [9], [29]. Chronic infection therefore maintains a constant cycle of inflammation, tissue damage, and repair that can also contribute to liver fibrosis. These cumulative effects may relate to the tendency for higher rates of IFN therapy-related toxicity and intolerance in co-infected patients. We did not consistently see evidence of interferon-associated leucopenia in co-infected patients, although HCV mono-infected patients had a significant reduction in WBC after interferon treatment without a decline in neutrophil percentage. Given the similar biomarker responses between mono- and co-infected patients after interferon treatment, it is difficult to determine whether certain biomarkers could account for these changes.

There were considerable changes to the cytokine profiles of co-infected patients who were successfully treated for HCV. Initially at BL, no markers were significantly different between C-SVR and C-NR patients; however, 15 were significantly different at FU, and the majority of these FU differences resulted from longitudinal changes in C-SVR patients. These same results were not seen in HCV mono-infected patients who achieved SVR, but this was likely due to the MST-SVR group’s smaller sample size.

All seven markers that increased significantly between BL and FU in C-SVR longitudinal analyses also became significantly higher in C-SVR than C-NR patients in the cross-sectional FU analysis. Four of these (IL-8, MCP-1, MIP-1β, and RANTES) are chemokines, suggesting heightened chemotactic activity in these patients, while the other three (PDGF-ββ, IL-7, and PAI-1) play important roles in angiogenesis, B and T cell development, and fibrinolysis inhibition, respectively. Interestingly, two of the chemokines, RANTES and MIP-1β, are both natural ligands for CCR5, a co-receptor used by HIV to bind and enter target cells. Several of the increased markers, including PDGF-ββ, IL-8, PAI-1, and MCP-1 are also thought to contribute to fibrosis and liver damage through various mechanisms [30]–[34]. We also found it interesting that MCP-1, in addition to other biomarkers, was significantly altered in several different analyses within our study. For example, MCP-1 significantly increased between BL and FU in the longitudinal analysis of C-SVR patients, was significantly higher in C-SVR patients than C-NR patients at FU, and was also significantly higher in co-infected versus mono-infected at BL. Most other studies of MCP-1 have either not evaluated or not found increased MCP-1 in HCV treatment responders compared to nonresponders, but most of these studies were limited to mono-infected populations [35]–[39]. Here we report that MCP-1 increased in co-infected patients who achieved an SVR and was significantly greater than in nonresponders at FU. Considering that MCP-1 plays a role in the chemotaxis of monocytes and their infiltration of the liver, has been associated with periportal inflammation and rapid HCV disease progression, and likely contributes to liver fibrosis, the MCP-1 results in our study deserve further investigation [35]–[36].

Four markers (IFN-α, M-CSF, sFasL, and TNF-β) decreased significantly between BL and FU in C-SVR patients and were also significantly lower in C-SVR than C-NR patients at FU. Considering that the majority of samples in this group were collected at week 56, patients demonstrated an end of treatment response (ETR), decreased levels of IFN-α indicated reduced antiviral activity after completing treatment and successfully eliminating the virus. Furthermore, significant decreases in sFasL and TNF-β among C-SVR patients may have interesting implications for liver pathology in this co-infected population since both sFasL and TNF-β can induce apoptosis and have been implicated in liver cell injury and disease progression [40]–[43]. A successful response to HCV treatment has been thought to cause a reduction in liver fibrosis, but our results demonstrate that more research is needed to confirm the role of these cytokines in the liver-healing process, and particularly in co-infected patients. Studies comparing successfully treated HCV patients to un-infected controls may help elucidate the role of these cytokines in regression of liver disease.

Our results indicate that cytokine profiles change considerably over the course of conventional HCV treatment in responders and differentiate SVR patients from NR at FU. These markers could help predict treatment responses earlier during therapy. Further studies to determine when IL-8, MCP-1, MIP-1β, PDGF-bb, IL-7, PAI-1, and RANTES levels first begin to increase or when M-CSF, sFasL, and TNF-β levels first begin to decrease in C-SVR patients could provide a better time line for predicting SVR.

Finally, we hypothesized that achieving SVR might curb the pro-inflammatory response, either partially or fully restoring inflammatory cytokine concentrations to levels comparable to our spontaneous clearance control group. However, results comparing the combined-SVR group and the spontaneous clearance group suggested that this was not the case. Twenty-two biomarkers were significantly elevated in the combined-SVR group at BL, and 12 remained significantly elevated at FU, indicating a continued inflammatory state. These results suggest that inflammation associated with chronic viral infection continues even after patients have achieved viral clearance, though we can only make this observation for samples taken shortly after treatment completion. Long-term biomarker responses after viral clearance are still unclear, and studies evaluating SVR patients at a later date could determine if or when cytokine profiles return to normal.

Our study was limited by a small sample size, and over 90% of the patients were male. We did not include HIV mono-infected or uninfected control groups to dissect out differences that could have been specific for HIV infection alone. Another limitation was that FU was limited to one time point, which varied among patients, as samples were taken around week 36 (in MST) or week 56 (in C-SVR and C-NR). Though samples were collected at different times, we considered them indicative of ETR since suppression of viremia, if observed, typically occurs much earlier between weeks four and twelve. In addition, duration of treatment varied for MST patients at the discretion of the provider, and several patients discontinued treatment due to ongoing medical conditions or treatment side effects. Limits of detection varied slightly across biomarker assay plates, which affected inter-assay comparison. However, similar results from analyses of mean fluorescence intensity (MFI), which is subject to less inter-plate variation, demonstrated that the impact of inter-assay variability was not significant (data not shown). Several patients in the C-SVR and C-NR groups (33% and 50% respectively) received G-CSF therapy for neutropenia at some point during IFN therapy, but subanalyses revealed that effects of this treatment were minimal (data not shown). Due to changes in the 50-plex assay available at the Stanford University Immune Monitoring Center, IL-18 results were not available for all samples and were thus were removed from the analysis. Our study only evaluated cytokine concentrations in the plasma, rather than localized cytokine expression in the liver where most HCV-related damage occurs and patterns could differ as a result of differences in cell tropism for these two viruses. Finally, we evaluated 50 different cytokines within each comparison. Accordingly, we used a strict significance level of 0.001 as a Bonferroni correction for 50 simultaneous tests. While this reduces the Type I error rate, it necessarily increases the Type II error rate. In addition, any non-randomized study is subject to potential confounding. We therefore considered models to adjust for potential confounders. Although biomarkers that were significant after correction for multiple comparisons in our initial model were still significant after adjustment, estimates under the adjusted model were unstable because of the limited sample size. Therefore, it is possible that some of our results are affected by clinical factors that may or may not have been measured in our study, although this issue is not unique to this study. However, considering these limitations, our results are important for showing general inflammation that likely contributes to the increased risk of comorbidities.

Further investigation of cytokine dynamics is warranted to understand the impact of HIV/HCV co-infection and HCV treatment on the pro-inflammatory response. More research is needed to determine which specific biomarker or cassette of biomarkers could serve as diagnostic tools for monitoring disease progression and treatment outcomes, which could improve therapeutic approaches. Future studies should include examination of earlier time points just after HCV treatment initiation, the impact of underlying host genetics (i.e. IL28b genotype), the effect of new HCV protease and polymerase inhibitors, and potential correlations between biomarker levels and histological or clinical degrees of liver fibrosis or dysfunction.

## Supporting Information

Appendix S1 **Expanded Cytokine and Chemokine Nomenclature (Alphabetical).** Alphabetical listing of all cytokines and chemokines referenced within the manuscript, as well as their expanded acronyms.
(DOC)

Appendix S2 **Baseline Analyses of Mono-infected, Co-infected, and HCV Spontaneous Clearance Patients.** Cross-sectional comparison of cytokine concentrations between (a) all mono-infected patients and all co-infected patients at baseline and (b) Cross-sectional comparison of cytokine concentrations between all mono-infected patients and HCV spontaneous clearance patients (designated HCV SC) at baseline; and between all co-infected patients and HCV SC patients at baseline. **†** p value: HCV SC group compared to all mono-infected**; ‡** p value: HCV SC group compared to all co-infected
(DOC)

Appendix S3 **Cross-sectional and Longitudinal Analyses of Baseline and Follow-Up Data for C-SVR and C-NR Cohorts.** Cross-sectional comparison of cytokine concentrations (conc.) between (a) co-infected patients who achieved an SVR (C-SVR) and co-infected patients who had a non-response (C-NR) at baseline (BL) and follow-up (FU). (b) Longitudinal comparison analyzing changes in cytokine concentration between baseline and follow-up within the C-SVR and C-NR cohorts.
(DOC)

Appendix S4 **Longitudinal Analyses of Baseline and Follow-Up Data for Groups that Deferred Treatment.** Longitudinal comparison analyzing changes in cytokine concentration between baseline and follow-up within the mono-infected group that deferred treatment (MDT) and the co-infected group that deferred treatment (CDT).
(DOC)

Appendix S5 **Baseline Comparison of Sustained Virologic Responders vs. Non-responders within the MST Group.** Cross-sectional comparison of cytokine concentrations between mono-infected patients who achieved a sustained virologic response (MST-SVR) and mono-infected patients who had a non-response (MST-NR) at baseline.
(DOC)

Appendix S6 **Longitudinal Analyses of MST Patients who Achieved SVR.** Longitudinal comparison analyzing changes in cytokine concentration between baseline and follow-up, within the mono-infected group that achieved a sustained virologic response (MST-SVR).
(DOC)

Appendix S7 **HCV Spontaneous Clearance Group versus Combined-SVR at Baseline and Follow-Up.** Cross-sectional comparisons between HCV spontaneous clearance patients (HCV SC) and i) all patients who achieved a sustained virologic response (Combined-SVR), ii) co-infected patients who achieved a sustained virologic response (C-SVR), and iii) mono-infected patients who achieved a sustained virologic response (MST-SVR). Data from the HCV SC group was compared to all three groups at both baseline (BL) and follow-up (FU).
(DOC)
