# Supplementary material for: Immune Biomarker Differences and Changes Comparing HCV Mono-Infected, HIV/HCV Co-Infected, and HCV Spontaneously Cleared Patients
Source: PLoS One. 2013 Apr 4;8(4):e60387. doi: 10.1371/journal.pone.0060387 (PMC3617231; doi:10.1371/journal.pone.0060387)
Supplement: Supplementary file 1 [file pone.0060387.s001.doc]

ENA78 (CXCL5)	Epithelial Neutrophil Activating Protein 78	IL-15	Interleukin-15
Eotaxin (CCL11)	-	IL-17	Interleukin-17
FGF-β	Fibroblast Growth Factor	IL-17F	Interleukin-17F
G-CSF	Granulocyte Colony-Stimulating Factor	IL-18	Interleukin-18
GM-CSF	Granulocyte Macrophage Colony-Stimulating Factor	Leptin	-
GRO-α (CXCL1)	Growth Related Oncogene Alpha	LIF	Leukemia Inhibitory Factor
HGF	Hepatocyte Growth Factor	MCP-1 (CCL2)	Monocyte Chemotactic Protein-1
ICAM-1	Intercellular Adhesion Molecule	MCP-3 (CCL7)	Monocyte Chemotactic Protein-3
IFN-α	Interferon-alpha	M-CSF	Macrophage Colony-Stimulating Factor
IFN-β	Interferon-beta	MIG (CXCL9)	Monokine Induced by IFN-gamma
IFN-γ	Interferon-gamma	MIP-1α (CCL3)	Macrophage Inflammatory Protein-1alpha
IP-10 (CXCL10)	Interferon-gamma-induced Protein-10	MIP-1β (CCL4)	Macrophage Inflammatory Protein-1beta
IL-1α	Interleukin-1 alpha	NGF	Nerve Growth Factor
IL-1β	Interleukin-1 beta	PAI-1	Plasminogen Activator Inhibitor- 1
IL-1RA	Interleukin-1 Receptor Antagonist	PDGF-ββ	Platelet Derived Growth Factor- Beta homodimer
IL-2	Interleukin-2	RANTES (CCL5)	Regulated on Activation Normal T Cell Expressed and Secreted
IL-4	Interleukin-4	Resistin	-
IL-5	Interleukin-5	SCF	Stem Cell Factor
IL-6	Interleukin-6	sFas Ligand	Soluble Fas Ligand
IL-7	Interleukin-7	TGF-α	Transforming Growth Factor-alpha
IL-8 (CXCL8)	Interleukin-8	TGF-β	Transforming Growth Factor-beta
IL-10	Interleukin-10	TNF-α	Tumor Necrosis Factor-alpha
IL-12p70	Interleukin-12p70	TNF-β	Tumor Necrosis Factor-beta (Lymphotoxin)
IL-12p40	Interleukin-12p40	VCAM-1	Vascular Cell Adhesion Molecule-1
IL-13	Interleukin-13	VEGF	Vascular Endothelial Growth Factor
