# Supplementary material for: Immune Biomarker Differences and Changes Comparing HCV Mono-Infected, HIV/HCV Co-Infected, and HCV Spontaneously Cleared Patients
Source: PLoS One. 2013 Apr 4;8(4):e60387. doi: 10.1371/journal.pone.0060387 (PMC3617231; doi:10.1371/journal.pone.0060387)
Supplement: Supplementary file 2 [file pone.0060387.s002.doc]

	(a)				(b)			
		Median Conc. (pg/ml)			Median Conc. (pg/ml)			
Function	Cytokine	Mono-infected	Co-infected	p-value		HCV SC	p-value †	p-value ‡
Inflammatory	IL-1α	4.9	16.4	**<0.001**		3.1	0.059	**<0.001**
	IL-1β	1.7	3.0	**<0.001**		2.1	0.217	0.004
	IL-6	1.2	4.1	**<0.001**		0.2	0.024	**<0.001**
	IL-12p40	5.8	29.1	**<0.001**		0.1	**<0.001**	**<0.001**
	IL-12p70	3.1	7.8	**<0.001**		2.0	**<0.001**	**<0.001**
	TNF-α	1.8	2.9	**<0.001**		0.7	**<0.001**	**<0.001**
Anti-inflammatory	IL-1RA	0.6	523.9	**<0.001**		366.1	**<0.001**	0.009
	IL-10	0.8	3.5	**<0.001**		0.2	0.072	**<0.001**
	TGF-β	0.1	1.7	**<0.001**		0.7	0.493	**<0.001**
Anti-viral	IFN-α	2.0	22.6	**<0.001**		0.0	**<0.001**	**<0.001**
	IFN-β	10.1	3.9	0.008		0.2	**<0.001**	**<0.001**
Chemokines	EOTAXIN (CCL11)	14.1	4.2	0.841		3.5	0.874	0.188
	ENA78 (CXCL5)	6.3	18.1	**<0.001**		0.1	**0.001**	**<0.001**
	GRO-α (CXCL1)	7.6	11.3	0.008		4.8	0.022	**<0.001**
	IL-8 (CXCL8)	2.5	1.4	**<0.001**		0.4	**<0.001**	**<0.001**
	IP-10 (CXCL10)	36.9	35.7	0.842		6.7	**<0.001**	**<0.001**
	MCP-1 (CCL2)	1.2	5.0	**<0.001**		3.1	**<0.001**	0.092
	MCP-3 (CCL7)	4.1	10.8	**<0.001**		2.7	0.069	**<0.001**
	MIG (CXCL9)	24.6	61.8	**<0.001**		6.8	0.003	**<0.001**
	MIP-1α (CCL3)	16.0	13.0	0.429		9.6	0.124	0.002
	MIP-1β (CCL4)	16.4	53.6	**<0.001**		27.6	0.019	0.006
	RANTES (CCL5)	367.5	250.4	0.270		38.4	**<0.001**	**<0.001**
Growth Factors	FGF-β	5.0	6.0	0.038		0.6	0.006	**<0.001**
	HGF	26.7	16.9	0.005		6.6	**<0.001**	**<0.001**
	NGF	5.0	7.0	0.565		3.8	0.238	**<0.001**
	PDGF-ββ	6.7	21.3	**<0.001**		1.2	**0.001**	**<0.001**
	TGF-α	3.7	4.8	0.078		0.1	**<0.001**	**<0.001**
	VEGF	4.0	3.7	0.442		0.1	**<0.001**	**<0.001**
Hematopoetic	IL-7	25.6	13.9	0.0011		0.0	**<0.001**	**<0.001**
	GM-CSF	5.6	10.0	0.007		5.4	0.720	0.040
	G-CSF	0.8	1.5	0.020		0.3	0.580	0.021
	M-CSF	6.2	13.2	**<0.001**		0.3	0.029	**<0.001**
	SCF	5.1	22.8	**<0.001**		6.4	0.416	**0.001**
Adaptive	IFN-γ	3.6	3.1	0.995		0.6	**<0.001**	**<0.001**
	IL-2	2.2	4.9	**0.001**		1.7	0.909	0.003
	IL-4	3.0	4.3	**<0.001**		2.1	0.006	**<0.001**
	IL-5	3.4	11.2	**<0.001**		1.3	0.002	**<0.001**
	IL-13	4.0	5.1	0.014		0.1	**<0.001**	**<0.001**
	IL-15	5.3	9.2	0.228		2.4	0.079	**0.000**
	IL-17	3.0	1.6	**<0.001**		0.3	**<0.001**	**<0.001**
	IL-17F	11.7	0.7	**<0.001**		0.0	**<0.001**	**<0.001**
Adipose Derived	Leptin	1491.5	446.2	0.002		282.0	0.010	0.387
	Resistin	1133.0	513.1	**<0.001**		180.7	**<0.001**	**<0.001**
CAMs	ICAM-1	80338.0	20203.1	0.749		2707.4	**0.001**	0.002
	VCAM-1	5104.7	7214.7	0.929		1593.5	**<0.001**	**<0.001**
Other	LIF	2.6	1.7	0.006		0.7	**<0.001**	**<0.001**
	PAI-1	432.9	293.1	0.221		57.1	**<0.001**	**<0.001**
	sFasL	3.3	35.5	**<0.001**		2.4	0.020	**<0.001**
	TNF-β	2.2	2.1	0.861		0.0	**<0.001**	**<0.001**
