# Supplementary material for: Immune Biomarker Differences and Changes Comparing HCV Mono-Infected, HIV/HCV Co-Infected, and HCV Spontaneously Cleared Patients
Source: PLoS One. 2013 Apr 4;8(4):e60387. doi: 10.1371/journal.pone.0060387 (PMC3617231; doi:10.1371/journal.pone.0060387)
Supplement: Supplementary file 3 [file pone.0060387.s003.doc]

		(a) Cross-Sectional Comparisons at Baseline and Follow-Up						(b) Longitudinal Comparison: Basline vs. Follow-Up					
		BL Median Conc. (pg/ml)			FU Median Conc. (pg/ml)			C-SVR			C-NR		
**Function**	**Cytokine**	**C-SVR**	**C-NR**	**p-value**	**C-SVR**	**C-NR**	**p-value**	Difference	Signed rank	**p value**	Difference	Signed rank	**p value**
Inflammatory	IL-1α	19.2	21.9	0.462	14.3	23.0	0.002	-4.27	-52.5	0.021	0.42	0.5	1.000
	IL-1β	3.0	4.0	0.054	2.6	3.9	**<0.001**	-0.43	-48.5	0.034	0.06	14.5	0.517
	IL-6	4.8	4.3	0.814	4.2	4.1	0.961	-1.30	-6	0.782	0.01	-13.5	0.548
	IL-12p40	35.1	37.1	0.605	21.0	45.9	**0.001**	-15.02	-67.5	0.002	3.24	39.5	0.064
	IL-12p70	8.1	9.2	0.187	10.9	7.9	0.752	2.20	27.5	0.246	-0.56	-20.5	0.353
	TNF-α	4.0	3.8	0.814	5.2	3.2	0.100	0.40	18.5	0.442	-0.04	-14.5	0.517
Anti-inflammatory	IL-1RA	568.6	543.5	0.583	474.5	556.3	0.005	-102.06	-65.5	0.003	0.32	7.5	0.747
	IL-10	3.6	4.1	0.142	2.2	4.5	0.007	-1.03	-31.5	0.145	0.24	1.5	0.963
	TGF-β	2.0	2.1	0.583	3.5	2.0	0.021	1.49	47.5	0.039	-0.14	-11.5	0.611
Anti-viral	IFN-α	24.7	24.7	0.638	10.8	28.7	**<0.001**	-14.94	-65	**<0.001**	1.59	28.5	0.190
	IFN-β	3.5	3.6	0.790	2.2	3.8	0.042	-1.14	-64.5	0.003	-0.51	-7.5	0.747
Chemokines	EOTAXIN (CCL11)	4.2	4.5	0.541	7.9	5.0	0.003	3.10	62.5	0.005	0.05	16.5	0.451
	ENA78 (CXCL5)	20.2	22.2	0.838	47.6	20.6	0.113	2.46	36.5	0.119	-0.22	-5.5	0.818
	GRO-α (CXCL1)	11.7	11.6	0.672	15.3	11.3	0.022	2.60	44.5	0.054	-0.11	-6	0.782
	IL-8 (CXCL8)	1.2	1.4	0.256	3.6	1.2	**<0.001**	2.33	66.5	**0.001**	-0.16	-43.5	0.040
	IP-10 (CXCL10)	25.1	35.4	0.390	23.5	33.2	0.024	-5.21	-32.5	0.167	-2.96	-9.5	0.678
	MCP-1 (CCL2)	6.5	6.0	0.425	13.9	5.2	**<0.001**	5.70	83.5	**<0.001**	0.39	14.5	0.517
	MCP-3 (CCL7)	10.6	14.6	0.060	12.1	15.6	0.002	-0.22	-12.5	0.610	2.13	25.5	0.244
	MIG (CXCL9)	106.9	61.3	0.077	79.4	85.5	0.845	-6.74	-22.5	0.347	13.25	29.5	0.174
	MIP-1α (CCL3)	12.9	14.3	0.425	17.9	15.5	0.166	4.17	51.5	0.013	-0.04	12.5	0.579
	MIP-1β (CCL4)	59.1	61.3	0.326	148.7	54.3	**<0.001**	87.08	71.5	**0.001**	-7.16	-39.5	0.064
	RANTES (CCL5)	196.9	181.4	0.583	685.4	223.3	**<0.001**	496.15	84.5	**<0.001**	19.52	17.5	0.431
Growth Factors	FGF-β	6.5	7.1	0.562	8.7	6.9	0.499	0.69	17.5	0.468	-0.24	-6.5	0.782
	HGF	14.0	18.4	0.168	21.0	14.9	0.030	5.58	46.5	0.043	-2.06	-29.5	0.174
	NGF	6.7	8.9	0.187	7.1	8.6	0.106	-0.17	-3.5	0.899	-0.12	-0.5	1.000
	PDGF-ββ	27.9	25.8	0.605	173.5	27.1	**<0.001**	126.68	81.5	**<0.001**	-0.79	2.5	0.927
	TGF-α	5.7	6.4	0.425	1.9	5.9	**0.001**	-4.75	-38.5	0.013	-0.38	-16.5	0.459
	VEGF	2.2	4.8	0.133	3.4	4.4	0.526	-0.20	-13	0.278	-1.67	-8.5	0.432
Hematopoetic	IL-7	13.3	10.6	0.533	38.0	11.3	**<0.001**	29.40	63	**<0.001**	0.12	-2	0.940
	GM-CSF	10.0	16.9	0.150	11.3	15.7	0.662	2.96	23.5	0.325	-0.11	-9.5	0.678
	G-CSF	1.6	2.3	0.157	3.8	2.1	0.032	2.31	62.5	0.005	0.48	22	0.229
	M-CSF	13.3	15.9	0.168	7.6	14.4	**<0.001**	-7.11	-73.5	**0.001**	-0.22	-5.5	0.818
	SCF	25.1	28.4	0.501	18.2	25.0	**0.001**	-7.10	-66.5	0.002	-0.52	-9.5	0.678
Adaptive	IFN-γ	3.1	3.7	0.243	3.4	3.4	0.619	-0.48	-19.5	0.417	-0.17	-11.5	0.611
	IL-2	6.0	6.3	0.742	11.4	5.0	0.007	4.74	36.5	0.119	-0.05	-9.5	0.678
	IL-4	4.3	5.7	0.142	3.7	5.7	0.003	-0.51	-63	0.004	0.18	-7	0.756
	IL-5	11.9	14.3	0.349	14.2	13.4	0.684	1.74	20.5	0.393	-0.42	-11.5	0.611
	IL-13	5.6	7.0	0.390	4.8	5.6	0.126	-1.03	-34.5	0.142	-0.81	-13.5	0.548
	IL-15	8.7	12.7	0.068	10.2	10.7	0.215	-0.76	-18.5	0.442	-3.90	-21.5	0.329
	IL-17	1.4	1.5	0.341	2.3	1.4	0.006	0.87	58.5	0.009	0.01	-12	0.587
	IL-17F	0.6	0.5	0.304	0.6	0.9	0.598	-0.04	-31	0.185	0.06	5.5	0.818
Adipose Derived	Leptin	409.7	443.2	0.838	416.0	367.4	0.641	59.04	15.5	0.523	-63.93	-33.5	0.120
	Resistin	445.4	507.4	0.425	450.8	595.8	0.003	-5.67	-18.5	0.442	56.59	33.5	0.120
CAMs	ICAM-1	29456.7	31394.5	0.546	5783.4	28725.4	0.009	-26334.26	-60	**<0.001**	1704.75	0	1.000
	VCAM-1	7297.5	7055.9	0.340	3778.4	5601.0	0.064	-3021.80	-84.5	**<0.001**	-2039.73	-1.5	0.500
Other	LIF	1.6	1.5	0.962	2.2	1.4	0.032	0.56	30.5	0.196	-0.12	-13.5	0.548
	PAI-1	284.7	312.2	0.373	806.6	337.9	**<0.001**	497.97	85.5	**<0.001**	2.20	-8.5	0.712
	sFasL	40.7	50.8	0.134	20.4	50.3	**<0.001**	-19.37	-77.5	**<0.001**	-0.63	-7.5	0.747
	TNF-β	2.2	2.4	0.672	0.8	2.1	**<0.001**	-1.43	-85.5	**<0.001**	-0.19	-38	0.073
