# Supplementary material for: Immune Biomarker Differences and Changes Comparing HCV Mono-Infected, HIV/HCV Co-Infected, and HCV Spontaneously Cleared Patients
Source: PLoS One. 2013 Apr 4;8(4):e60387. doi: 10.1371/journal.pone.0060387 (PMC3617231; doi:10.1371/journal.pone.0060387)
Supplement: Supplementary file 4 [file pone.0060387.s004.doc]

		CDT			MDT		
**Function**	**Cytokine**	Difference	Signed rank	**p value**	Difference	Signed Rank	**p-value**
Inflammatory	IL-1α	0.3	5.0	0.55	0.39	15.5	0.131
	IL-1β	0.0	-6.0	0.46	-0.06	-6	0.572
	IL-6	0.0	-5.0	0.13	0.00	-0.5	1.000
	IL-12p40	0.1	6.0	0.46	0.17	10.5	0.309
	IL-12p70	-0.1	-6.0	0.45	0.02	4	0.719
	TNF-α	0.0	2.0	0.84	-0.11	-7.5	0.492
Anti-inflammatory	IL-1RA	0.0	-1.5	0.50	0.00	1.5	0.844
	IL-10	-0.1	-11.5	0.12	0.01	4.5	0.637
	TGF-β	0.0	.	.	0.00	.	.
Anti-viral	IFN-α	0.0	4.0	0.64	0.02	2.5	0.846
	IFN-β	0.1	-1.0	0.94	-0.10	-0.5	1.000
Chemokines	EOTAXIN (CCL11)	0.9	3.5	0.44	0.00	-1	0.750
	ENA78 (CXCL5)	0.0	-1.5	0.50	0.00	0	1.000
	GRO-α (CXCL1)	0.2	0.0	1.00	0.84	16.5	0.106
	IL-8 (CXCL8)	0.0	0.0	1.00	0.03	11.5	0.264
	IP-10 (CXCL10)	10.7	5.0	0.55	15.79	20.5	0.037
	MCP-1 (CCL2)	0.0	.	.	0.00	.	.
	MCP-3 (CCL7)	0.0	-2.0	0.81	0.22	6.5	0.557
	MIG (CXCL9)	-0.3	-2.0	0.84	1.71	11.5	0.275
	MIP-1α (CCL3)	0.0	0.5	1.00	0.21	5.5	0.313
	MIP-1β (CCL4)	2.4	5.0	0.47	0.00	6.5	0.219
	RANTES (CCL5)	-215.9	-17.0	0.02	91.74	13.5	0.193
Growth Factors	FGF-β	0.0	0.5	1.00	0.00	4	0.578
	HGF	0.8	3.0	0.74	3.35	8.5	0.432
	NGF	1.0	4.5	0.44	0.00	2	0.813
	PDGF-ββ	-6.9	-14.0	0.05	3.53	16.5	0.106
	TGF-α	0.0	-1.5	0.50	0.00	7.5	0.156
	VEGF	0.1	2.0	0.84	0.97	19.5	0.020
Hematopoetic	IL-7	-2.8	-4.0	0.64	10.15	14.5	0.160
	GM-CSF	-0.1	-2.0	0.84	1.47	15.5	0.131
	G-CSF	0.0	-1.5	0.84	0.08	5.5	0.625
	M-CSF	0.1	0.0	1.00	0.03	6.5	0.496
	SCF	-0.1	0.0	1.00	0.20	9.5	0.375
Adaptive	IFN-γ	-0.1	-4.0	0.58	0.28	6.5	0.557
	IL-2	0.0	0.0	1.00	0.00	-0.5	1.000
	IL-4	0.0	-2.5	0.77	-0.01	3.5	0.770
	IL-5	0.2	10.0	0.20	0.64	22.5	0.020
	IL-13	0.0	-4.0	0.64	0.00	2	0.865
	IL-15	0.0	-1.0	0.75	0.00	-3.5	0.563
	IL-17	-0.1	-3.0	0.74	0.03	9.5	0.375
	IL-17F	0.3	1.0	0.94	-0.37	-0.5	1.000
Adipose Derived	Leptin	195.2	11.0	0.15	492.55	17	0.016
	Resistin	366.5	8.0	0.31	137.64	9.5	0.375
CAMs	ICAM-1	0.0	.	.	0.00	0.5	1.000
	VCAM-1	0.0	-1.5	0.50	0.00	1.5	0.844
Other	LIF	0.1	1.0	0.95	0.25	13.5	0.193
	PAI-1	43.8	5.0	0.55	180.73	18.5	0.065
	sFasL	-0.7	-18.0	0.01	0.25	14.5	0.160
	TNF-β	0.0	2.0	0.81	0.01	14	0.170
