# Supplementary material for: Immune Biomarker Differences and Changes Comparing HCV Mono-Infected, HIV/HCV Co-Infected, and HCV Spontaneously Cleared Patients
Source: PLoS One. 2013 Apr 4;8(4):e60387. doi: 10.1371/journal.pone.0060387 (PMC3617231; doi:10.1371/journal.pone.0060387)
Supplement: Supplementary file 5 [file pone.0060387.s005.doc]

		Median Concentration (pg/ml)		
**Function**	**Cytokine**	**MST- SVR**	**MST-NR**	**p value**
Inflammatory	IL-1α	4.9	4.9	1.00
	IL-1β	1.8	1.7	0.58
	IL-6	1.2	1.2	0.92
	IL-12p40	8.3	6.7	0.63
	IL-12p70	3.3	3.1	0.49
	TNF-α	1.7	2.1	0.36
Anti-inflammatory	IL-1RA	2.2	2.9	0.94
	IL-10	1.1	0.8	0.40
	TGF-β	0.1	0.3	0.66
Anti-viral	IFN-α	2.7	2.5	1.00
	IFN-β	8.7	17.8	0.45
Chemokines	EOTAXIN (CCL11)	26.2	55.4	0.73
	ENA78 (CXCL5)	8.3	12.7	0.20
	GRO-α (CXCL1)	9.9	10	0.53
	IL-8 (CXCL8)	2.6	3.1	0.16
	IP-10 (CXCL10)	45	55.7	0.63
	MCP-1 (CCL2)	1.2	1.2	0.91
	MCP-3 (CCL7)	4.6	5.3	0.66
	MIG (CXCL9)	27.4	26.4	0.73
	MIP-1α (CCL3)	17.9	20.1	0.45
	MIP-1β (CCL4)	19.1	30.3	0.78
	RANTES (CCL5)	407.9	353.4	0.83
Growth Factors	FGF-β	5.5	6	0.63
	HGF	25.2	96.5	0.16
	NGF	6.3	10.8	0.73
	PDGF-ββ	6.7	7.2	0.78
	TGF-α	4.1	5	0.34
	VEGF	4.3	5.6	0.43
Hematopoetic	IL-7	45.2	29	0.79
	GM-CSF	5.6	4.9	0.83
	G-CSF	0.5	1	0.20
	M-CSF	7.8	8.1	0.81
	SCF	5.5	5.9	0.63
Adaptive	IFN-γ	3.9	3.3	0.63
	IL-2	2.1	2.8	0.53
	IL-4	3.1	3	0.53
	IL-5	2.8	4	0.20
	IL-13	4.2	4.1	0.81
	IL-15	3.4	6.3	0.53
	IL-17	3.1	3.2	1.00
	IL-17F	8.1	20.9	0.26
Adipose Derived	Leptin	1271	1527.9	0.45
	Resistin	790.2	1625.7	0.06
CAMs	ICAM-1	45523	80338	0.82
	VCAM-1	16981	6152.3	0.94
Other	LIF	2.6	2.7	0.83
	PAI-1	251.7	472.2	0.53
	sFasL	5.9	4.8	0.83
	TNF-β	2.4	2.4	0.94
