# Supplementary material for: Immune Biomarker Differences and Changes Comparing HCV Mono-Infected, HIV/HCV Co-Infected, and HCV Spontaneously Cleared Patients
Source: PLoS One. 2013 Apr 4;8(4):e60387. doi: 10.1371/journal.pone.0060387 (PMC3617231; doi:10.1371/journal.pone.0060387)
Supplement: Supplementary file 6 [file pone.0060387.s006.doc]

		MST- SVR		
**Function**	**Cytokine**	Difference	Signed rank	**p value**
Inflammatory	IL-1α	-0.7	-7.5	0.16
	IL-1β	-0.3	-7.5	0.16
	IL-6	0.0	-1.5	0.50
	IL-12p40	-0.3	-1.5	0.81
	IL-12p70	-0.2	-4.0	0.47
	TNF-α	-0.5	-6.5	0.22
Anti-inflammatory	IL-1RA	-2.2	-10.5	0.03
	IL-10	-0.5	-10.5	0.03
	TGF-β	0.0	.	.
Anti-viral	IFN-α	2.8	4.5	0.44
	IFN-β	-2.1	-2.5	0.69
Chemokines	EOTAXIN (CCL11)	-0.3	-3.5	0.44
	ENA78 (CXCL5)	-9.9	-7.5	0.06
	GRO-α (CXCL1)	-3.4	-10.5	0.03
	IL-8 (CXCL8)	-0.5	-10.5	0.03
	IP-10 (CXCL10)	-7.1	-7.5	0.16
	MCP-1 (CCL2)	0.0	0.5	1.00
	MCP-3 (CCL7)	-0.5	-4.5	0.31
	MIG (CXCL9)	-5.8	-4.5	0.44
	MIP-1α (CCL3)	-11.9	-10.5	0.03
	MIP-1β (CCL4)	-6.1	-2.5	0.63
	RANTES (CCL5)	-110.2	-8.5	0.09
Growth Factors	FGF-β	-3.8	-10.5	0.03
	HGF	-4.8	-4.5	0.44
	NGF	-2.7	-7.5	0.16
	PDGF-ββ	-3.3	-6.5	0.22
	TGF-α	-1.9	-3.0	0.25
	VEGF	-0.6	-5.5	0.19
Hematopoetic	IL-7	-4.5	-2.5	0.63
	GM-CSF	-0.3	-2.5	0.69
	G-CSF	-0.3	-3.0	0.25
	M-CSF	-6.6	-10.5	0.03
	SCF	-1.4	-7.5	0.16
Adaptive	IFN-γ	-1.2	-6.5	0.22
	IL-2	-1.3	-10.5	0.03
	IL-4	-0.2	-6.5	0.22
	IL-5	-0.9	-9.5	0.06
	IL-13	-0.5	-4.5	0.44
	IL-15	-1.2	-6.5	0.22
	IL-17	-0.6	-8.5	0.09
	IL-17F	-2.1	-4.5	0.31
Adipose Derived	Leptin	-280.1	-6.5	0.22
	Resistin	-116.6	-7.5	0.16
CAMs	ICAM-1	-4189.2	-3.0	0.25
	VCAM-1	-7451.9	-5.0	0.13
Other	LIF	-0.1	-4.0	0.47
	PAI-1	1.0	-1.5	0.84
	sFasL	-3.9	-10.5	0.03
	TNF-β	-0.7	-10.5	0.03
