# Supplementary material for: Immune Biomarker Differences and Changes Comparing HCV Mono-Infected, HIV/HCV Co-Infected, and HCV Spontaneously Cleared Patients
Source: PLoS One. 2013 Apr 4;8(4):e60387. doi: 10.1371/journal.pone.0060387 (PMC3617231; doi:10.1371/journal.pone.0060387)
Supplement: Supplementary file 7 [file pone.0060387.s007.doc]

		BL Median Conc. (pg/ml)		BL p-values			FU Median Conc. (pg/ml)	FU p-values		
Function	Cytokine	HCV SC	Combined-SVR	HCV SC vs. Combined-SVR	HCV SC vs. C-SVR	HCV SC vs. MST-SVR	Combined-SVR	HCV SC vs. Combined-SVR	HCV SC vs. C-SVR	HCV SV vs. MST-SVR
Inflammatory	IL-1α	3.14	13.44	**<0.001**	**<0.001**	0.2347	11.14	**<0.001**	**<0.001**	0.4702
	IL-1β	2.12	2.43	0.082	0.003	0.6768	2.22	0.332	0.0132	0.1085
	IL-6	0.15	4.16	**<0.001**	**<0.001**	**<0.001**	3.27	**<0.001**	**<0.001**	0.0460
	IL-12p40	0.05	23.37	**<0.001**	**<0.001**	**<0.001**	17.05	**<0.001**	**<0.001**	**<0.001**
	IL-12p70	1.98	5.68	**<0.001**	**<0.001**	**<0.001**	7.40	**<0.001**	**<0.001**	**<0.001**
	TNF-α	0.70	2.62	**<0.001**	**<0.001**	**<0.001**	4.23	**<0.001**	**<0.001**	0.0293
Anti-inflammatory	IL-1RA	366.08	501.14	0.267	**<0.001**	0.0143	400.19	0.732	0.0031	0.0031
	IL-10	0.24	2.66	**<0.001**	**<0.001**	0.0021	1.83	**0.001**	**<0.001**	0.1313
	TGF-β	0.66	1.24	0.012	**<0.001**	0.3694	3.31	**0.001**	**<0.001**	0.5376
Anti-viral	IFN-α	0.01	20.73	**<0.001**	**<0.001**	**<0.001**	9.28	**<0.001**	**<0.001**	**<0.001**
	IFN-β	0.17	4.28	**<0.001**	**<0.001**	**<0.001**	3.37	**<0.001**	**<0.001**	0.0040
Chemokines	EOTAXIN (CCL1	3.50	4.32	0.132	0.4468	0.1537	7.91	0.002	**<0.001**	0.6216
	ENA78 (CXCL5)	0.08	17.19	**<0.001**	**<0.001**	**<0.001**	22.10	**<0.001**	**<0.001**	0.1136
	GRO-α (CXCL1)	4.75	11.27	**<0.001**	**<0.001**	0.0033	12.24	**<0.001**	**<0.001**	1.00
	IL-8 (CXCL8)	0.35	1.44	**<0.001**	**<0.001**	**<0.001**	2.60	**<0.001**	**<0.001**	0.0022
	IP-10 (CXCL10)	6.74	32.12	**<0.001**	**<0.001**	**<0.001**	25.29	**<0.001**	**<0.001**	**<0.001**
	MCP-1 (CCL2)	3.11	3.38	0.606	0.0148	0.0352	9.80	0.027	**<0.001**	0.1968
	MCP-3 (CCL7)	2.69	9.25	**<0.001**	**<0.001**	0.1146	10.59	**0.001**	**<0.001**	0.4861
	MIG (CXCL9)	6.84	59.93	**<0.001**	**<0.001**	0.0033	59.22	**<0.001**	**<0.001**	0.0184
	MIP-1α (CCL3)	9.61	15.94	**<0.001**	**<0.001**	**<0.001**	16.10	0.003	**<0.001**	0.2044
	MIP-1β (CCL4)	27.56	46.45	0.041	**<0.001**	0.2051	108.66	0.004	**<0.001**	0.173
	RANTES (CCL5)	38.43	250.44	**<0.001**	**<0.001**	0.0024	564.27	**<0.001**	**<0.001**	0.0046
Growth Factors	FGF-β	0.56	6.18	**<0.001**	**<0.001**	**<0.001**	6.74	**<0.001**	**<0.001**	**<0.001**
	HGF	6.55	19.15	**<0.001**	**<0.001**	**<0.001**	22.27	**<0.001**	**<0.001**	0.0062
	NGF	3.79	6.77	**<0.001**	**<0.001**	0.0944	7.04	0.002	**<0.001**	0.9096
	PDGF-ββ	1.15	17.98	**<0.001**	**<0.001**	0.0281	111.67	**<0.001**	**<0.001**	0.0913
	TGF-α	0.09	5.05	**<0.001**	**<0.001**	**<0.001**	2.01	**<0.001**	**<0.001**	**<0.001**
	VEGF	0.09	3.56	**<0.001**	**<0.001**	**<0.001**	3.55	**<0.001**	**<0.001**	**<0.001**
Hematopoetic	IL-7	0.04	15.84	**<0.001**	**<0.001**	**<0.001**	35.97	**<0.001**	**<0.001**	**<0.001**
	GM-CSF	5.40	8.31	0.129	0.0246	1.00	10.93	0.034	**<0.001**	0.6104
	G-CSF	0.32	1.16	0.043	0.0307	0.5935	3.39	0.007	**<0.001**	0.0715
	M-CSF	0.35	11.26	**<0.001**	**<0.001**	**<0.001**	5.69	**0.001**	**<0.001**	0.5539
	SCF	6.42	18.76	0.006	**<0.001**	0.8955	14.82	0.012	**<0.001**	0.3318
Adaptive	IFN-γ	0.61	3.35	**<0.001**	**<0.001**	**<0.001**	3.43	**<0.001**	**<0.001**	0.0011
	IL-2	1.70	4.66	**0.001**	**<0.001**	0.5693	9.55	0.015	**<0.001**	0.0434
	IL-4	2.14	3.34	**<0.001**	**<0.001**	0.0233	3.20	**<0.001**	**<0.001**	0.2051
	IL-5	1.27	7.49	**<0.001**	**<0.001**	0.0337	9.49	**<0.001**	**<0.001**	0.0894
	IL-13	0.12	4.90	**<0.001**	**<0.001**	**<0.001**	4.11	**<0.001**	**<0.001**	**<0.001**
	IL-15	2.41	8.19	**0.001**	**<0.001**	0.4129	9.29	0.002	**<0.001**	0.778
	IL-17	0.26	1.59	**<0.001**	**<0.001**	**<0.001**	2.52	**<0.001**	**<0.001**	**<0.001**
	IL-17F	0.00	0.89	**<0.001**	**<0.001**	**<0.001**	0.74	**<0.001**	**<0.001**	**<0.001**
Adipose Derived	Leptin	282.02	569.35	0.141	0.413	0.4702	489.54	0.296	0.532	0.9699
	Resistin	180.68	511.11	**<0.001**	0.0013	**<0.001**	488.82	**<0.001**	**<0.001**	0.0062
CAMs	ICAM-1	2707.40	43298.95	**0.001**	0.0024	0.0168	5783.39	0.100	0.4056	0.0894
	VCAM-1	1593.49	7261.45	**<0.001**	**<0.001**	**<0.001**	4453.21	**<0.001**	**<0.001**	0.0032
Other	LIF	0.74	2.01	**<0.001**	**<0.001**	0.0058	2.34	**<0.001**	**<0.001**	0.0173
	PAI-1	57.13	290.11	**<0.001**	**<0.001**	0.0033	770.20	**<0.001**	**<0.001**	0.0084
	sFasL	2.42	26.87	**<0.001**	**<0.001**	0.0084	14.53	**0.001**	**<0.001**	0.8500
	TNF-β	0.01	2.36	**<0.001**	**<0.001**	**<0.001**	1.09	**<0.001**	**<0.001**	**<0.001**
